# Medication-related harm in New Zealand general practice: a retrospective records review

**DOI:** 10.3399/BJGP.2020.1126

**Published:** 2021-06-29

**Authors:** Sharon Leitch, Susan M Dovey, Wayne K Cunningham, Alesha J Smith, Jiaxu Zeng, David M Reith, Katharine A Wallis, Kyle S Eggleton, Andrew W McMenamin, Martyn I Williamson, Steven Lillis

**Affiliations:** Department of General Practice and Rural Health, Otago Medical School, Dunedin Campus, University of Otago, Dunedin, New Zealand.; Department of General Practice and Rural Health, Otago Medical School, Dunedin Campus, University of Otago, Dunedin, New Zealand.; Department of Family Medicine, Royal College of Surgeons in Ireland, Medical University of Bahrain (RCSI Bahrain), Adliya, Bahrain.; School of Pharmacy, University of Otago, Dunedin, New Zealand.; Department of Preventive and Social Medicine, Otago Medical School, Dunedin Campus, University of Otago, Dunedin, New Zealand.; Otago Medical School, Dunedin Campus, University of Otago, Dunedin, New Zealand.; University of Queensland, Brisbane, Australia.; Faculty of Medical and Health Sciences, University of Auckland, Auckland, New Zealand.; Te Ngae Medical Centre, Rotorua, New Zealand.; Department of General Practice and Rural Health, Otago Medical School, Dunedin Campus, University of Otago, Dunedin, New Zealand.; Faculty of Medical and Health Sciences, University of Auckland, Auckland, New Zealand.

**Keywords:** general practice, New Zealand, patient harm, primary health care, retrospective studies

## Abstract

**Background:**

The extent of medication-related harm in general practice is unknown.

**Aim:**

To identify and describe all medication-related harm in electronic general practice records. The secondary aim was to investigate factors potentially associated with medication-related harm.

**Design and setting:**

Retrospective cohort records review study in 44 randomly selected New Zealand general practices for the 3 years 2011–2013.

**Method:**

Eight GPs reviewed 9076 randomly selected patient records. Medication-related harms were identified when the causal agent was prescribed in general practice. Harms were coded by type, preventability, and severity. The number and proportion of patients who experienced medication-related harm was calculated. Weighted logistic regression was used to identify factors associated with harm.

**Results:**

In total, 976 of 9076 patients (10.8%) experienced 1762 medication-related harms over 3 years. After weighting, the incidence rate of all medication-related harms was 73.9 harms per 1000 patient–years, and the incidence of preventable, or potentially preventable, medication-related harms was 15.6 per 1000 patient–years. Most harms were minor (*n* = 1385/1762, 78.6%), but around one in five harms were moderate or severe (*n* = 373/1762, 21.2%); three patients died. Eighteen study patients were hospitalised; after weighting this correlates to a hospitalisation rate of 1.1 per 1000 patient–years. Increased age, number of consultations, and number of medications were associated with increased risk of medication-related harm. Cardiovascular medications, antineoplastic and immunomodulatory agents, and anticoagulants caused most harm by frequency and severity.

**Conclusion:**

Medication-related harm in general practice is common. This study adds to the evidence about the risk posed by medication in the real world. Findings can be used to inform decision making in general practice.

## BACKGROUND

Reducing medication-related harm is a top priority for improving patient safety.^1^^,^^2^ Primary healthcare settings remain relatively unexamined for patient harm.^3^ It is possible patient harm in general practice has been underestimated.^4^ Medication-related harm accounts for around 3% of all hospital admissions on average, with higher rates observed in older people.^5^^–^^8^

Clinical trials, event reporting, and compensation claims provide a limited perspective on medication-related harm in the real world, producing data not typically generalisable to general practice populations. Population-based records review research can identify harms experienced in the course of routine clinical care and identify patients at increased risk of harm to improve patient safety.^9^

This study examined medication-related harm in general practice using a subset of data from a nation-wide retrospective cohort review of general practice electronic health records that looked at all harms.^10^^,^^11^ The primary aim of this study was to estimate the incidence, preventability, and severity of all harms attributable to medication prescribed in general practice in New Zealand. The secondary aim was to investigate factors potentially associated with medication-related harm, including age, sex, ethnicity, social deprivation, number of consultations, number of medications, and general practice size and location.

## METHOD

### Setting

All New Zealand general practices were stratified by size and location.^10^^,^^11^ Practice size was defined by the number of enrolled patients, divided into tertiles to form three groups consisting of large, medium, and small practices. Location was defined as rural or urban based on the practice address.^10^^,^^11^ Practice size and location defined six strata. Twelve practices were randomly selected from each strata and invited to participate; 44 study practices consented to participate (71.0% of the 62 eligible randomly selected practices with compatible practice software).^11^

**Table:** How this fits in

The extent of medication-related harm in general practice is unknown. This retrospective records review found that medication-related harm in general practice is common, and is typically minor and arising from standard care. Patients who are older, who have more consultations, and who take more medication are at greatest risk of harm. The risk of patient harm increased with age. Patients aged 60–74 years had nearly double the risk of harm compared with the reference group (patients aged 15–59 years), and patients aged >75 years had triple the risk. This knowledge can inform shared decision making about treatment options.

### Participants

Patients enrolled in recruited practices were randomly selected for participation at the mid-point of the study period; in total, 9076 patients were randomly selected (based on prior power calculations).^11^ The general practice records of the randomly selected patients for the 3-year study period (1 January 2011 to 31 December 2013, inclusive) were anonymised at the time of electronic data extraction. The extracted records contained everything that is normally available in patient records, including demographic data, consultation notes, screening data, laboratory and radiology results, referral letters, alerts, and prescriptions. Secondary care referrals, discharge summaries, and clinic letters were available where these had been stored electronically in the record.

Consent and data access were granted by each practice, rather than from individual patients.^12^ This research was approved by the University of Otago ethics committee, and reviewed by the Ngāi Tahu Research Consultation Committee.

### Reviewers

Each patient’s file was examined by at least one of eight clinically active GPs, each with a minimum of 10 years’ experience. Reviewers participated in training sessions at the commencement of the study. Feedback from double-reviewed files (*n* = 948, 10.4%) was used to further improve reviewer consistency. The range of agreement between pairs of reviewers was 66.7%–100.0%; overall kappa = 0.344, *P*<0.001.

### Covariates

Patient demographic data including age at 1 July 2012, sex, self-identified ethnicity,^13^ and socioeconomic deprivation were obtained. Mãori are the indigenous people of New Zealand. Pasifika refers to the people of the Pacific islands (for example, Samoa, Tonga, and so on) who are now living in New Zealand. Participants were sorted into one of five socioeconomic categories, ranging from 1 (least deprived) to 5 (most deprived) based on their home address and census-derived data for each area meshblock.^14^ Information on the number of unique medications prescribed and number of consultations were obtained within the specified period. Practice size and location are defined above.

### Outcomes

Harm was defined as: *‘physical, emotional or financial negative consequences to patients directly arising from health care, beyond the usual consequences of care, and not attributable to patients’ health conditions.’* ^15^ Reviewers identified episodes where patients experienced harm, as documented in their records. Other patient safety measures, such as ‘near-misses’, ‘safety incidents’, ‘inappropriate prescribing’, and ‘errors’, were not recorded unless they resulted in patient harm. Each patient record was recorded in binary terms: harm or no harm.

Harm was rated minor, moderate, severe, or death.^10^ Short-lived and relatively trivial harms were coded as minor (for example, rashes, vomiting, and inconvenience to patients, such as being given the wrong prescription). Moderate harm was defined as having increased or persistent morbidity (for example, fractures, untreated anaemia, and poor diabetic control). Severe harms included renal failure, pulmonary embolism, myocardial infarction, and morphine overdose. Reviewers used their clinical expertise to assess preventability from five categories.^10^^,^^16^ Following discussion and consensus these options were aggregated in analysis to ‘preventable or potentially preventable’ (original codes: ‘preventable and originated in primary care’ and ‘potentially preventable and originated in primary care‘) and ‘not preventable’ (‘not preventable, standard treatment’, ‘not preventable and originated in primary care’, ‘not preventable and originated in secondary care’, and ‘preventable and originated in secondary care, or not preventable and originated in primary care’).

**Figure 1.**
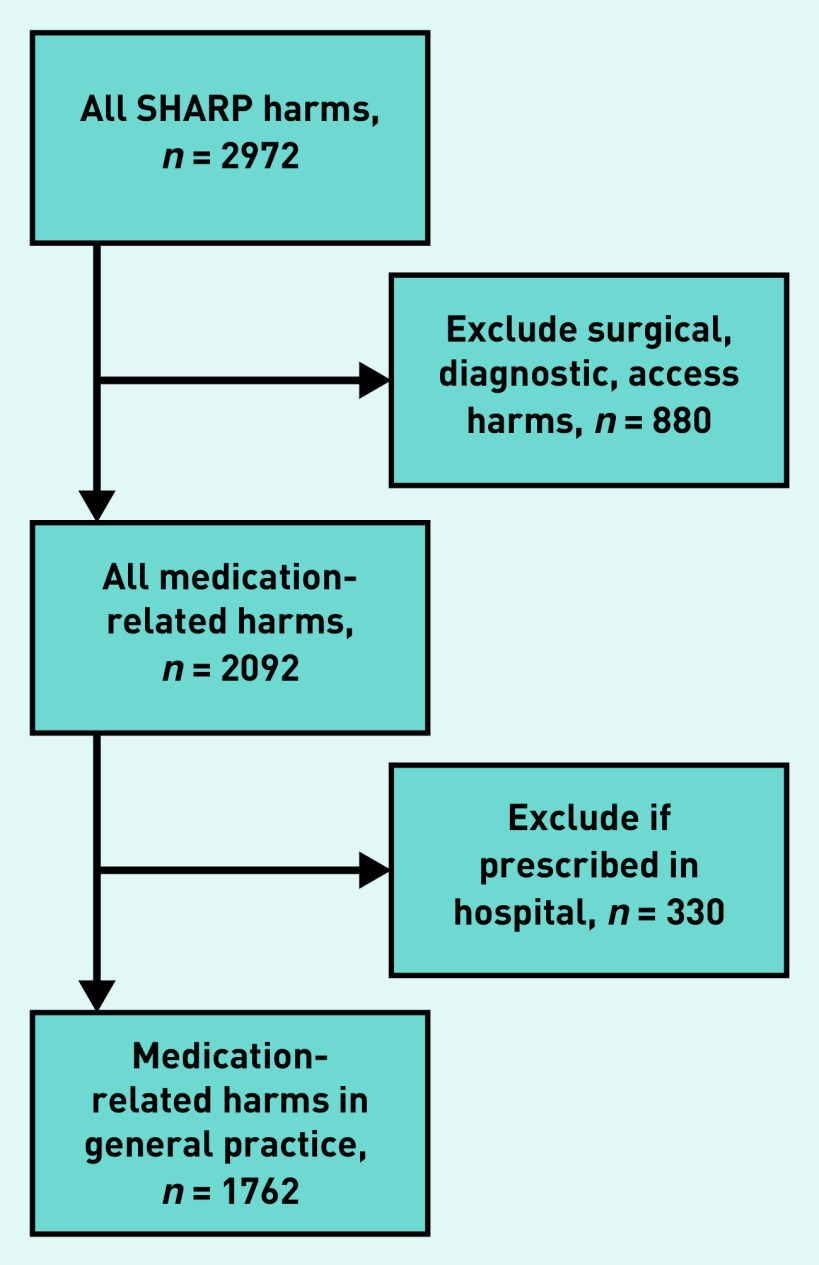
*Selection of medication-related harms data from records review study data. SHARP = Safety, Harms and Risk Reduction Project.*

Harms were documented in descriptive form, then coded using *Medical Dictionary for Regulatory Activities 18.0* codes.^17^ Data extraction is depicted in Figure 1. Medications were coded by drug type, using the Anatomical Therapeutic Chemical (ATC) classification system.^18^

### Statistical Analysis

The number and proportion of medication-related harms was calculated by patient demography (age, sex, ethnicity, and deprivation), clinical information (number of consultations and number of unique medications prescribed during the study period), and practice characteristics (practice size and location). Incidence rates were calculated as the number of events divided by the total number of person–years of follow-up (for example, 3*9076 years, 3 years per person). In order to obtain an estimate of the incidence of medication-related harm in New Zealand, sampling weights were applied to the incidence rates allowing for the probability of each practice being selected per strata, and each patient being selected per practice. Harms were examined by ATC classification. Individual medications were examined by rate of prescribing and percentage of patients harmed.

Logistic regression with robust standard error was used to explore associations between medication-related harm and patient demographics, clinical information, and practice characteristics. The final model included all covariates listed above. Estimates were then adjusted using appropriate sampling weights.

Stata (version 15.1) was used for all statistical analyses. The Stata svy package was used for applying sample weights. Data were missing for ethnicity (*n* = 139, 1.5%) and deprivation (*n* = 894, 9.9%). Complete data analyses were carried out on 8053 patients.

## RESULTS

From 2011–2013 inclusive, 7308 of 9076 (80.5%) patients received 175 657 prescriptions for 846 different medications from their general practices; 1770 (19.5%) patients were not prescribed any medications. Patients were prescribed 0–53 different medications each (median 4 [IQR 1–9]). Reviewers identified 1762 medication-related harms in 976 (10.8%) patient records over the 3-year study period: 255 different medications were associated with harm. Medication-related harm accounted for 59.0% of all 2972 harms observed in the record review study. After applying weighting, the incidence rate of medication-related harm in New Zealand general practice was 73.9 harms per 1000 patient–years, and the incidence rate of preventable or potentially preventable medication-related harm was 15.6 harms per 1000 patient–years (Table 1). Table 1 outlines the relationship between medication-related harms, patient demographics, clinical variables (numbers of consultations and medications), and practice characteristics as unweighted data and weighted estimates. Table 2 presents the logistic regression models of study variables in relation to medication-related harm.

**Table 1.** Demographic data of study patients, clinical exposure, and pratices in relation to medication-related harm related to GP prescribing

	**Unweighted study data, *n* (%)**		**Weighted data, *n* (%)^a^**	
	
	**No harm**	**Medicine-related harm**	**No harm**	**Medicine-related harm**
**Total**	8100/9076 (89.2)	976/9076 (10.8)	3 737 889/4 240 293 (88.2)	502 404/4 240 293 (11.8)

**Age, years**				
0–4	296 (94.6)	17 (5.4)	146 698 (93.0)	11 114 (7.0)
5–14	1283 (97.6)	32 (2.4)	599 128 (96.5)	21 511 (3.5)
15–59	4765 (93.2)	345 (6.8)	2 274 914 (92.1)	195 620 (7.9)
60–74	1217 (80.0)	305 (20.0)	504 945 (76.7)	153 736 (23.3)
>75	539 (66.1)	277 (33.9)	212 204 (63.8)	120 424 (36.2)

**Sex**				
Female	4189 (87.8)	583 (12.2)	1 972 810 (86.9)	298 012 (13.1)
Male	3911 (90.9)	393 (9.1)	1 765 079 (89.6)	204 392 (10.4)

**Ethnicity^b^**				
European	6092 (88.4)	797 (11.6)	2 901 377 (87.1)	428 700 (12.9)
Māori	1207 (91.0)	119 (9.0)	385 728 (90.2)	42 081 (9.8)
Pasifika	298 (94.3)	18 (5.7)	102 189 (95.0)	5322 (5.0)
Other	384 (94.6)	22 (5.4)	306 709 (93.7)	20 675 (6.3)

**Deprivation^c,d^**				
1	1762 (89.6)	204 (10.4)	1 165 530 (88.5)	150 861 (11.5)
2	1655 (88.9)	207 (11.1)	829 358 (87.4)	119 827 (12.6)
3	1525 (89.7)	176 (10.3)	663 132 (89.2)	79 880 (10.8)
4	1202 (88.8)	152 (11.2)	469 926 (87.0)	70 324 (13.0)
5	1149 (88.5)	150 (11.5)	385 526 (87.1)	57 219 (12.9)

**Number of consultations**				
0–3	2466 (99.7)	8 (0.3)	1 081 613 (99.5)	5567 (0.5)
4–12	3096 (95.9)	132 (4.1)	1 476 184 (94.5)	86 466 (5.5)
>13	2538 (75.2)	836 (24.8)	1 180 091 (74.2)	410 371 (25.8)

**Number of medications**				
0–4	4601 (98.6)	64 (1.4)	2 115 238 (98.0)	43 101 (2.0)
5–9	2099 (89.1)	257 (10.9)	956 015 (87.3)	139 267 (12.7)
>10	1400 (68.1)	655 (31.9)	666 636 (67.6)	320 036 (32.4)

**Practice size**				
Large	2650 (88.2)	353 (11.8)	2 409 416 (87.0)	358 999 (13.0)
Medium	2729 (88.6)	351 (11.4)	927 812 (89.6)	107 132 (10.4)
Small	2721 (90.9)	272 (9.1)	400 661 (91.7)	36 273 (8.3)

**Practice location**				
Urban	4082 (89.8)	462 (10.2)	3 050 365 (88.0)	416 372 (12.0)
Rural	4018 (88.7)	514 (11.3)	687 524 (88.9)	86 032 (11.1)

a *Weighting was applied based on the relative probability of each practice being selected per strata, and each person being selected to participate per practice, due to the complex sampling design of the study. Weighting means these results are nationally generalisable to the New Zealand population.*

b *Missing data = 139.*

c *Missing data = 894.*

d *Deprivation is based on New Zealand Index of Deprivation (socioeconomic deprivation), where 1 = least deprived, and 5 = most deprived.^14^*

**Table 2.** Logistic regression of study variables in relation to harms arising from medication prescribed in general practice (binary outcome variables medication-related harm: harm or no harm)

**Variable**	**Unadjusted^a^**		**Adjusted^b^**		**Adjusted and weighted^c^**	
		
	**OR (95% CI)**	***P*-value**	**OR (95% CI)**	***P* -value**	**OR (95% CI)**	***P*-value**
**Age, years**						
0–4	0.79 (0.48 to 1.31)	0.365	0.56 (0.31 to 1.00)	0.049	0.75 (0.42 to 1.33)	0.308
5–14	0.34 (0.24 to 0.50)	<0.001	0.60 (0.41 to 0.88)	0.010	0.58 (0.31 to 1.10)	0.095
15–59	Reference	—	Reference	—	Reference	—
60–74	3.46 (2.93 to 4.09)	<0.001	1.81 (1.49 to 2.19)	<0.001	1.98 (1.50 to 2.61)	<0.001
>75	7.10 (5.92 to 8.51)	<0.001	2.86 (2.30 to 3.56)	<0.001	3.08 (2.15 to 4.41)	<0.001

**Sex**						
Male	Reference	—	Reference	—	Reference	—
Female	1.39 (1.21 to 1.59)	<0.001	1.07 (0.91 to 1.26)	0.397	0.98 (0.68 to 1.43)	0.931

**Ethnicity**						
European	Reference	—	Reference	—	Reference	—
Māori	0.75 (0.62 to 0.92)	0.006	1.03 (0.81 to 1.32)	0.790	1.01 (0.81 to 1.27)	0.924
Pasifika	0.46 (0.29 to 0.75)	0.002	0.57 (0.33 to 0.96)	0.036	0.43 (0.19 to 0.98)	0.045
Other	0.44 (0.29 to 0.69)	<0.001	0.86 (0.52 to 1.42)	0.554	0.68 (0.41 to 1.15)	0.145

**Deprivation^d^**						
1	Reference	—	Reference	—	Reference	—
2	1.08 (0.88 to 1.33)	0.459	1.00 (0.80 to 1.27)	0.969	1.04 (0.79 to 1.37)	0.783
3	1.00 (0.81 to 1.23)	0.977	0.92 (0.72 to 1.18)	0.528	0.86 (0.58 to 1.29)	0.457
4	1.09 (0.87 to 1.36)	0.437	1.05 (0.82 to 1.36)	0.685	1.15 (0.80 to 1.65)	0.443
5	1.13 (0.90 to 1.41)	0.292	1.14 (0.87 to 1.49)	0.360	1.05 (0.58 to 1.90)	0.871

**Number of consultations**						
0–3	Reference	—	Reference	—	Reference	—
4–12	13.14 (6.43 to 26.88)	<0.001	6.18 (2.77 to 13.77)	<0.001	5.38 (1.55 to 18.67)	0.009
>13	101.54 (50.50 to 204.16)	<0.001	15.21 (6.74 to 34.34)	<0.001	11.83 (4.27 to 32.80)	<0.001

**Number of medications**						
0–4	Reference	—	Reference	—	Reference	—
5–9	8.80 (6.66 to 11.63)	<0.001	3.41 (2.45 to 4.74)	<0.001	3.05 (2.10 to 4.44)	<0.001
>10	33.63 (25.84 to 43.78)	<0.001	7.25 (5.19 to 10.11)	<0.001	5.71 (3.83 to 8.50)	<0.001

**Practice size**						
Large	Reference	—	Reference	—	Reference	—
Medium	0.97 (0.83 to 1.13)	0.662	0.91 (0.75 to 1.10)	0.336	0.72 (0.46 to 1.11)	0.134
Small	0.75 (0.64 to 0.89)	<0.001	0.75 (0.61 to 0.93)	0.008	0.65 (0.44 to 0.95)	0.027

**Practice location**						
Urban	Reference	—	Reference	—	Reference	—
Rural	1.13 (0.99 to 1.29)	0.071	0.92 (0.78 to 1.08)	0.203	0.78 (0.55 to 1.09)	0.145

a *Unadjusted: unweighted univariate logistic regression.*

b *Adjusted: unweighted multiple logistic regression to adjust for potential confounders — all other variables were considered potential confounders.*

c *Adjusted and weighted: multiple logistic regression weighted for the relative probability of each person being selected as a study participant.*

d *Deprivation is based on New Zealand Index of Deprivation (socioeconomic deprivation), where 1 = least deprived, and 5 = most deprived.^14^ OR = odds ratio.*

### Patients

Older patients were more likely to experience medication-related harm. In the final model (adjusted and weighted) patients aged 60–74 years had double the odds of experiencing medication-related harm (odds ratio [OR] 1.98, 95% confidence interval [CI] = 1.50 to 2.61), and patients aged >75 years had triple the odds (OR 3.08, 95% CI = 2.15 to 4.41), compared to patients aged 15–59 years.

Women appeared to be at increased risk of medication-related harms in the unadjusted model; however, after adjustment for the other variables there was no difference in risk by sex. The smallest ethnic group was Pasifika (*n* = 316, 3.5%), which had a lower risk of experiencing harm than Europeans (OR 0.43, 95% CI = 0.19 to 0.98) (Table 2). There was no evidence that social deprivation was associated with medication-related harm.

### Clinical exposure

Increasing number of consultations and medications were correlated with increased risk of medication-related harm. Compared to patients who had 0–3 consultations over the study period, the odds of experiencing medication-related harm for patients that had 4–12 consultations over the 3-year study period was 5.38 (95% CI = 1.55 to 18.67) times greater; for patients with >13 consultations over 3 years the odds were 11.83 (95% CI = 4.27 to 32.80) times greater (Table 2). Similarly, when compared with patients prescribed 0–4 unique medications in the study period, being prescribed 5–9 medications was associated with an increased OR of medication-related harm of 3.05 (95% CI = 2.10 to 4.44). Being prescribed >10 medications was associated with an increased OR of 5.71 (95% CI = 3.83 to 8.50) (Table 2).

### Practices

Practice size was associated with risk of medication-related harm, but practice location was not. Patients attending small practices had a lower OR of experiencing medication-related harm compared to patients attending large practices (OR for patients attending small-sized practices 0.65, 95% CI = 0.44 to 0.95; OR for patients attending medium-sized practices 0.72, 95% CI = 0.46 to 1.11) (Table 2).

### Harms

Most medication-related harm was directly related to the medication (*n* = 1673/1762, 94.9%), but 5.1% was attributable to indirect causes such as access, communication (for example, asthma deteriorated as patient did not understand fluticasone needed to be taken regularly), or procedures (for example, local pain and swelling following administration of vaccine). Gastroenterological effects were the most common harm type by body system (*n* = 387, 22.0%) (Table 3). Medication-related harms were mainly of minor severity (*n* = 1385, 78.6%) (for example, angiotensin-converting enzyme inhibitor cough). Most medication-related harms were not preventable (*n* = 1432, 81.3%) (for example, weight gain with oral contraceptive); the remainder were considered preventable or potentially preventable (*n* = 330, 18.7%) (for example, cardiac arrest following co-prescription of medications that increased the QT interval). one in five harms were moderate or severe (*n* = 373/1762, 21.2%) (for example, developed type 2 diabetes following a long-term course of prednisone) or severe (*n* = 44, 2.5%) (for example, ventricular tachycardia and cardiac arrest attributed to amiodarone causing prolongation of the the QT interval), and four harms were associated with the deaths of three patients (*n* = 4, 0.2%). Eighteen patients were hospitalised as a result of medication-related harm, representing 0.2% (*n* = 18/9076) of all study patients and corresponding to a weighted hospitalisation rate of 1.1 per 1000 patient–years.

**Table 3.** Harm types by system with examples, *N* = 1762

**System, *n* (%)**	**Harm, *n* (% of system)**		**Examples**
**General** 186 (10.6)	Generally unwell	63 (33.9)	75-year-old female felt dizzy and sleepy after taking donepezil. Mild severity, not preventable.
	Fatigue	47 (25.3)	
	Weight change	24 (12.9)	10-year-old male experienced anorexia and poor weight gain on methylphenidate. Mild severity, potentially preventable.
	Exacerbation of existing condition	20 (10.8)	
	Other	32 (17.2)	

**Gastroenterology** 387 (22.0)	Nausea, vomiting, and diarrhoea	213 (55.0)	2-year-old female developed diarrhoea after takingamoxicillin. Mild severity, not preventable.
	Constipation	53 (13.7)	81-year-old male developed severe constipation from codeine requiring hospitalisation. Severe harm, potentially preventable.
	Dyspepsia	48 (12.4)	
	Bleeding	28 (7.2)	
	Pain	12 (3.1)	
	Other	33 (8.5)	

**Cardiology** 217 (12.3)	Hypotension	136 (62.7)	93-year-old male experienced recurrent falls secondary to hypotension while taking cilazapril, metoprolol, frusemide, and isosorbide mononitrate. Moderate severity, potentially preventable.
	Heart failure	39 (18.0)	
	Arrhythmias	27 (12.4)	
	Other	15 (6.9)	

**Neurology** 192 (10.9)	Cognition	61 (31.8)	83-year-old female experinced a haemorrhagic cerebrovascular accident after commencing aspirin and clopidogrel, resulting in death. Not preventable.
	Sensory	41 (21.4)	
	Headache	35 (18.2)	
	Balance	32 (16.7)	79-year-old male developed postural hypotension while taking metoprolol and cilazapril. Fell and developed a subdural haemaotoma, died during hospitalisation. Potentially preventable.
	Movement	12 (6.3)	
	Intracerebral event	11 (5.7)	

**Renal** 161 (9.1)	Renal	139 (86.3)	69-year-old male with severe chronic renal failure died within 2 weeks of an increased dose of metformin and allopurinol. Death, potentially preventable.
	Urology	22 (13.7)	

**Musculoskeletal** 107 (6.1)	Pain	75 (70.1)	81-year-old male experienced repeated episodes of gout while taking bendrofluazide. Mild severity, potentially preventable.
	Gout	18 (16.8)	
	Bones and joints	14 (13.1)	

**Skin** 104 (5.9)	Rash	50 (48.1)	8-year-old female developed scalp irritation and discomfort after using malathion shampoo. Mild severity, not preventable.
	Itch	23 (22.1)	
	Other	31 (29.8)	

**Mental health** 101 (5.7)	Mood/affect	66 (65.3)	53-year-old male experienced vivid dreams and sleep disturbance while taking varenicline. Mild severity, not preventable.
	Sleep disturbance	26 (25.7)	
	Addiction	9 (8.9)	43-year-old female described as abusing prescribed codeine. Moderate severity, potentially preventable.

**Haematology** 81 (4.6)	Haematology	77 (95.1)	49-year-old male developed thrombocytopenia while taking carbamazebine. Mild severity, not preventable.
	Immunology	4 (4.9)	

**Endocrine** 71 (4.0)	Diabetes related	48 (67.6)	71-year-old female taking glipizide and insulin experienced recurrent hypoglycaemic episodes. Moderate severity, potentially preventable.
	Sweating and flushing	10 (14.1)	
	Other	13 (18.3)	

**Reproductive health** 60 (3.4)	Bleeding	34 (56.7)	51-year-old female on dabigatran experienced menorrhagia requiring a blood transfusion. Moderate severity, not preventable.
	Infection/discharge	18 (30.0)	
	Pregnancy	8 (13.3)	

**Respiratory** 57 (3.2)	Cough and wheeze	57 (100)	71-year-old male developed acute pneumonitis while taking amiodarone. Severe harm, not preventable.

**Economic** 38 (2.2)	Extra treatment required	38 (100)	32-year-old male required hospitalisation and time off work for a gastrointestinal bleed while taking diclofenac and no proton-pump inhibitor. Moderate severity, potentially preventable.

### Medications

Table 4 shows harm by ATC classification group. Harms from cardiovascular medications (ATC Group C), predominantly antihypertensives and statins, affected the most patients; 517 patients were harmed of 5965 patients prescribed those medications (8.7%); 2.1% (*n* = 11/517) of those harms were severe. Antineoplastic and immunomodulatory agents (ATC Group L) had the highest rate of harm (*n* = 21/131, 16.0%) but none of the harms were severe, and these agents were taken by only 1.4% (*n* = 131/9076) of patients. Medication relating to blood and blood forming organs (ATC Group B) were the third most harmful agents affecting 6.0% (*n* = 102/1688) of study patients taking those medications, the most harmful being dabigatran (B01AE07), warfarin (B01AA03), and dipyridamole (B01AC07). This group had the highest proportion of severe harms (6.9%, *n* = 7/102). Analgesia, antibiotics, and asthma medications were among the most commonly prescribed medication types. Of these commonly prescribed medications, diclofenac and amoxicillin with clavulanic acid were associated with the most harm (*n* = 27/1016, 2.7%, and *n* = 21/926, 2.3%, respectively).

**Table 4.** Medication-related harm by ATC classification group

**ATC classification group**		**Percentage of patients harmed/patients prescribed unique medicine, *n* (%), *n* = 1433/55 340 (2.6%)^a^**	**Percentage of patients harmed as a proportion of medication-related harm by ATC class, *n* = 1433/1433 (100%)**
A	**Alimentary tract and metabolism**	124/6174 (2.0)	8.7
B	**Blood and blood forming organs**	102/1688 (6.0)	7.1
C	**Cardiovascular system**	517/5956 (8.7)	36.1
D	**Dermatologicals**	25/6385 (0.4)	1.7
G	**Genitourinary system and sex hormones**	52/1482 (3.5)	3.6
H	**Systemic hormonal preparations**	30/1653 (1.8)	2.1
J	**Anti-infectives for systemic use**	152/10 676 (1.4)	10.6
L	**Antineoplastic and immunomodulating agents**	21/131 (16.0)	1.5
M	**Musculoskeletal system**	91/4600 (2.0)	6.4
N	**Nervous system**	291/9178 (3.2)	20.3
P	**Antiparasitics, insecticides, and repellents**	4/377 (1.1)	0.3
R	**Respiratory system**	16/5612 (0.3)	1.1
S	**Sensory organs**	7/1330 (0.5)	0.5
V	**Various**	1/98 (1.0)	0.1

a *Each unique medicine was counted once per patient. Patients may have been prescribed* >*1 medicine in each ATC code; therefore, the total may be* >*100% of study patients in some categories. ATC = Anatomical Therapeutic Chemical.*

## DISCUSSION

### Summary

The incidence rate of medication-related harm in New Zealand general practice after weighting was 73.9 harms per 1000 patient–years; the incidence rate of potentially preventable medication-related harm was 15.6 harms per 1000 patient–years. Most medication-related harms were of minor severity, but three patients died. The hospitalisation rate was 1.1 per 1000 patient-years. Factors strongly associated with medication-related harm were increasing age and clinical exposure. Pasifika ethnicity and attending a small practice were protective. Cardiovascular medications caused the most harm.

### Strengths and limitations

General practice records are a rich data source, permitting comprehensive review of medication-related harms.^9^ The authors believe this large, detailed, retrospective review of a nationally representative sample of general practice records is likely to provide the closest possible estimate of medication-related harm in the real world. Harm rates are generalisable to the entire country. There have been few appreciable changes in New Zealand general practice prescribing since the study period, although medication use and polypharmacy have increased slightly.^19^^,^^20^

Harm rates presented should be considered a conservative estimate. Only recorded harms are included; it is unknown how many additional harms occurred but were not recorded. The authors assume all patient participants selected at the midpoint of the study period remained enrolled for the 3-year study period. Medication-related harms were only included if there was a prescription for the corresponding agent in the electronic medical record. Therefore, harms arising from medications administered or dispensed in general practice without a prescription (for example, some contraceptives or practitioner supply medications^21^) were not included. Additionally, controlled drugs, such as morphine and methylphenidate, required a hand-written prescription during the study period, but a concurrent electronic prescription may not have been generated. Harms were recorded verbatim — for example, it is not possible to know whether someone would have experienced haematemesis regardless of whether they had been taking diclofenac.

Harm estimates are not easily comparable between studies due to variations in terminology and methodology.^22^^–^^24^ Critics of the record review method point to this and object to low rates of reproducibility.^25^^,^^26^ However, the records review method is comprehensive and provides unique insight into the patient experience of medication-related harm.^9^ Reviewer training and feedback were used to improve reviewer concordance.

### Comparison with existing literature

The authors’ research found medication-related harm was common, for several reasons. Records were examined for all medication-related harm, and not just preventable adverse events or patient-safety incidents; the patient-focused definition of harm was comprehensive; and the authors examined all patient records (not just those considered high risk or identified by a trigger tool). The figures are therefore higher than published figures, although comparisons between these types of studies are difficult. The most comparable systematic review estimated the incidence of preventable adverse drug events as 15 per 1000 person-years,^23^ which is equivalent to the incidence rate for preventable or potentially preventable medication-related harm. Other studies indicate medication-related harm is a substantial problem, but are less comparable with the findings of this study. One meta-analysis found up to 24 patient safety incidents per 100 primary care consultations, with up to 11% of medication-related incidents resulting in patient harm;^27^ a literature review found up to 2.3% of deaths followed adverse events attributable to primary care treatment, with up to 42% of serious medication-related harms in primary care considered preventable;^28^ while a record review study found 25.7% of preventable harms attributable to medication.^29^

### Implications for research and practice

General practice has been considered a relatively safe healthcare setting. This study found medication-related harm is common in general practice, mostly minor, not preventable, and often arising from standard care. However, sometimes harms resulted in severe outcomes including hospitalisation and death; one in four harms were considered at least potentially preventable. These findings reinforced the need for vigilance and care in even routine medication use.

This research adds to the field’s knowledge of which patients are at highest risk of medication-related harm; namely, patients who are older, who have more consultations, and who take more medications. Identifying these patients may help inform shared decision making at the time of prescribing and target risk monitoring. Further research is required to determine how best to address and reduce the risk of medication-related harm in the context of routine general practice prescribing.

Medication-related harm in general practice is common. This study builds on the evidence base about the risk posed by medication in the real world. Findings can be used to inform decision making in general practice and to target patient safety initiatives towards patients at higher risk of harm.
